# Nanoporous Gold: Fabrication, Characterization, and Applications

**DOI:** 10.3390/ma2042188

**Published:** 2009-12-03

**Authors:** Erkin Seker, Michael L. Reed, Matthew R. Begley

**Affiliations:** 1 Center for Engineering in Medicine, Department of Surgery, Harvard Medical School, Massachusetts General Hospital, Shriners Hospitals for Children, Boston, MA 02114, USA; 2 Department of Electrical and Computer Engineering, University of Virginia, Charlottesville, VA 22904, USA; E-Mails: reed@virginia.edu (M.L.R.); begley@virginia.edu (M.R.B.); 3 Department of Mechanical and Aerospace Engineering, University of Virginia, Charlottesville, VA 22904, USA; 4 Department of Materials Science and Engineering, University of Virginia, Charlottesville, VA 22904, USA

**Keywords:** nanoporous, gold, microfabrication, characterization, surface area, morphology, MEMS, catalyst, sensor, gold-silver alloy

## Abstract

Nanoporous gold (np-Au) has intriguing material properties that offer potential benefits for many applications due to its high specific surface area, well-characterized thiol-gold surface chemistry, high electrical conductivity, and reduced stiffness. The research on np-Au has taken place on various fronts, including advanced microfabrication and characterization techniques to probe unusual nanoscale properties and applications spanning from fuel cells to electrochemical sensors. Here, we provide a review of the recent advances in np-Au research, with special emphasis on microfabrication and characterization techniques. We conclude the paper with a brief outline of challenges to overcome in the study of nanoporous metals.

## 1. Introduction

There exists a large inventory of porous materials with a variety of pore sizes and morphologies to benefit numerous applications. The requirements of each application and basic scientific curiosity demand innovative processing and characterization techniques to understand the fundamental properties of these materials. A subset of porous materials is porous metals, which are of special interest due to their catalytic activity, electrical conductivity, and mechanical properties. Among porous metals, nanoporous gold (np-Au) has attracted distinct attention from the scientific community and industry alike. This surge of interest is evidenced by the recent increase in the number of papers published on np-Au: the ~25 papers appearing in the thirteen years spanning 1992–2005 has blossomed into more than 80 in the last four years (2006–2009). Controllable pore morphology of np-Au provides a highly adaptable system for the fundamental study of wide range of mechanical and surface properties [1]. Despite the growing number of studies on various aspects of np-Au, such as its catalytic and optical applications [2], there are still many under-explored features of this material, including surface functionalization via thiol-conjugate chemistry and biological applications.

The purpose of this review is to provide a brief highlight of np-Au research, followed by discussion of fabrication and characterization methods. We hope that this review will serve as a survey of general current knowledge on np-Au, as well as provide a more focused look at current fabrication techniques and characterization methods to study the relationship between material and mechanical properties.

## 2. Research Fronts

**Figure 1:**
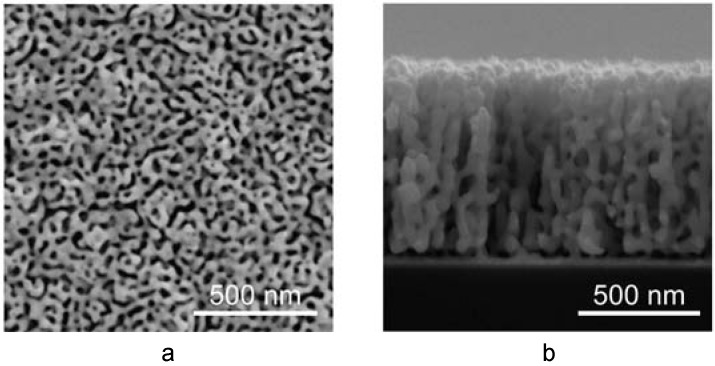
Scanning electron micrographs of typical np-Au morphology: (a) top view, and (b) cross-sectional view.

While the main research fronts in np-Au overlap in many ways, it is possible to categorize them, more or less, under the following headings: fabrication, characterization, and applications. The first two categories will be the main focus of this review. In this section, we intend to briefly explore the latter along with several nanoscale phenomena, but first, let us briefly describe np-Au. Selective dissolution, also known as leaching or dealloying, is a corrosion process that is routinely used to produce porous metals, including np-Au. This process entails the removal of a less noble constituent of an alloy in a strong corrosive environment, where the surface diffusion of the noble constituent produces an open pore network structure that consists mostly of the noble constituent. Figure 1 is a scanning electron micrograph illustrating the typical top and cross-sectional morphologies of np-Au, which reveal its homogenous and inter-connected pore structure.

### 2.1. Sensor Applications

It is not surprising that high surface area-to-volume ratio of np-Au has been its primary feature that inspired various sensor applications. For instance, Hieda *et al.* increased the sensitivity of quartz crystal microbalance gas sensors 40-fold by replacing the typical gold electrodes with np-Au [3]. Lavrik *et al.* focused on chemo-mechanical deformations of cantilevers due to surface stress changes caused by adsorption of biomolecules (*i.e.,* protein A and biotinilyated albumin) on atomic force microscope (AFM) beam surfaces coated with planar gold and np-Au films [4]. They reported that 50–75 nm-thick np-Au films resulted in several microns of deformation, which constituted a nearly order of magnitude improvement over deformations attained with planar gold coatings. Liu *et al.* used np-Au nanowires to monitor resistance change upon adsorption of monolayer of octadecanethiol [5]. The authors obtained a sensitivity factor comparable to ultrathin films by exploiting the dominance of surface scattering effects due to feature size of np-Au smaller than the mean free path for electrons in bulk gold. Hu *et al.* developed a DNA biosensor with 28 attomolar detection limit by first immobilizing single-stranded DNA on the nanoporous surface, then consecutively hybridizing it to target DNA, and finally hybridizing it to reporter DNA attached to a gold nanoparticle [6]. Shulga *et al.* detected concentrations down to 0.1 ng/mL of prostate specific antigens by immobilized capture antibodies labeled with alkaline phosphatase on electroplated np-Au electrodes [7]. Mortari *et al.* utilized the increased electrochemical double layer capacitance, which correlates with increased surface area, to detect milk fouling in terms of capacitive signal changes with 30-fold enhancement compared to planar gold [8]. Yang *et al.* coated np-Au films with platinum for amperometric detection of Escherichia coli measurement with a detection limit of 10 cfu/mL [9]. Liu *et al.* demonstrated the potential of np-Au as an electrochemical sensor for p-nitrophenol, a pollutant in waste waters [10]. Zhu *et al.* encapsulated cytochrome c in np-Au films preserving its enzymatic activity and demonstrated its potential as an amperometric H_2_O_2_ sensor [11]. For the first time, the functionality of np-Au with live tissue was demonstrated by successful detection of field potentials from organotypic hippocampal slices on np-Au multiple electrode arrays (MEAs). Using this method, the authors were able to reduce electrode impedance by more than 25-fold compared to planar gold electrodes, thereby enabling sensitive measurements in electrically noisy tissue culture incubators [12]. In general substituting traditional sensor materials with np-Au gold offered improved detection limits and signal-to-noise ratios. We envision that the multiplexing and miniaturization of such sensors, accompanied by their integration with signal processing circuitry, may lead to the development of point-of-care devices for important applications, including sensitive viral load measurements and blood panels. Microfabrication, as it will be discussed later in this review, will be instrumental in achieving these goals.

### 2.2. Surface- and Porosity-Related Phenomenon

The nanoscale porosity and high surface area of np-Au leads to fascinating phenomena, which most probably can only be witnessed in such length-scales and morphologies. Chemo-mechanical activity of np-Au, for instance, was attributed the dominance of surface area over total volume, exemplified by elastic deformation of np-Au under an electrical potential or exposure to unstable gases (e.g., carbon monoxide and ozone) [13,14]. Unusually high reversible elastic contraction of millimeter size np-Au samples during the anodic part of cyclic potential scans suggested that the underlying mechanism may be a monolayer of oxygen adsorption and/or ultra small ligament size (1–2 nm), that is, ultra-high surface area [15]. On a different front, np-Au surfaces, with their highly tunable roughness, serve as platforms for surface enhancement Raman spectroscopy (SERS) and can facilitate detection of adsorbed molecules with high sensitivity. Two different groups demonstrated strong enhancement of Raman scattering on np-Au surfaces and attributed this to electromagnetic SERS enhancement due to field localization within pores [16,17]. Regarding biomolecule-surface interaction, grafting density of DNA molecules not only increased on np-Au films (compared to planar gold), but also a strong DNA strand-length dependence of grafting density was evident [18]. Here, it was suggested that the adsorption of DNA inside the pores caused steric hindrance against the adsorption of subsequent molecules with a non-linear dependence on strand lengths over ten base pairs. This phenomenon may find use in development of size-dependent sorting of biomolecules by creating pore-size gradients. Yet another unique phenomenon lies at the intersection of nanoscale porosity and fluid mechanics, wherein the central observation is that a liquid drop on a np-Au film forms a wetting “halo” around its periphery, in which the size of the halo remains constant independent of droplet size. An experimentally validated model describing the wetting phenomenon as a balance between surface energy, viscous loss (as fluid flows from the droplet into the film) and evaporative loss captured physical basis of this phenomenon [19]. Currently, the aforementioned phenomena appeal mostly to fundamental studies in order to understand the underlying mechanisms; however, we expect that the findings in this area will pave the way to innovative applications in the near future.

### 2.3. Catalysis

The enhanced catalytic activity and electrical conductance of np-Au encouraged several groups to engineer catalytic platforms. Zhang *et al.* demonstrated increased oxidation of methanol on np-Au films compared to planar gold, suggesting that trapping of OH- anions in the porous network greatly facilitates oxidation [20]. Similarly, Xu *et al.* used np-Au as a catalytic platform for enhanced oxidation of CO [21]. Although gold displays reasonable catalytic activity, platinum is required for certain applications such as fuel cells due to its higher catalytic efficiency. Zeis *et al.* offered a solution to this necessity by uniformly electroplating thin layers of platinum on the surface of a np-Au foil, thereby using np-Au as a structural template, reducing the platinum consumption and its associated expense [22]. They expanded this technique to construct Pt-coated np-Au foil/Nafion membranes for fuel cell applications and generated power densities up to 4.5 kW/g, with comparable performance to conventional fuel cells. Catalytic applications employing np-Au are in their infancy, but we expect that demonstration of large-scale production of such catalysts will promote technology development.

## 3. Fabrication and Synthesis Methods

The desire to utilize np-Au in various applications, as well as fundamental studies, demand the development of novel fabrication and synthesis techniques: many novel approaches have emerged recently. This section discusses the underlying mechanisms of porosity formation, methods to produce alloys and the final nanoporous structure, techniques to control pore morphology, and microfabrication strategies to incorporate np-Au in microsystems.

### 3.1. Porosity Formation

**Figure 2:**
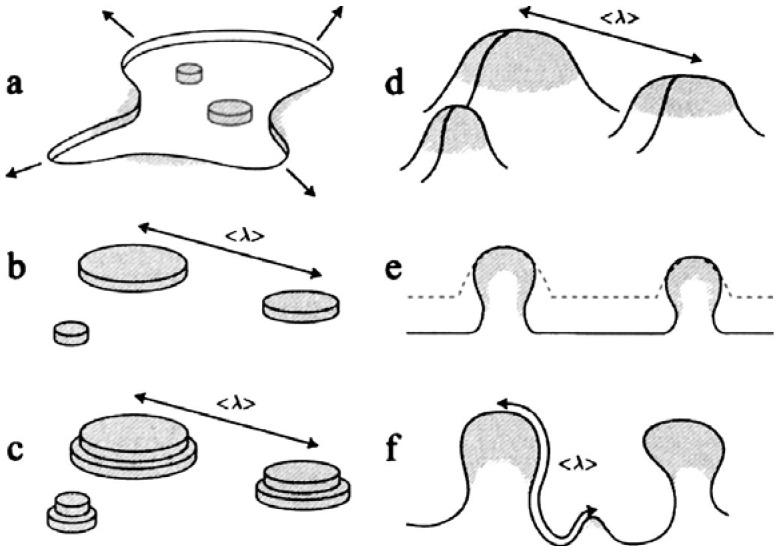
Illustration of porosity formation during dealloying: (a) lateral removal of less noble atoms (uncolored) leading to clustering of noble atoms (shaded) on surface; (b) supplied with remaining noble atoms from dissolution, clusters coarsen until the next alloy layer is attacked, as the characteristic length *<λ>* appears; (c) noble atom-capped hills form as the second layer of alloy dissolves, while the characteristic length between hills remains the same; (d) less noble atoms can accumulate at the bases of hills, since their perimeters are widening, without changing the characteristic length; (e) undercutting of hills (compared the original morphology denoted with a dashed line) and increase in average distance between hills measured along the alloy-electrolyte interface; (f) new noble atom hills nucleate as the hill-hill distance along the alloy-electrolyte distance is approximately twice the characteristic length. [Reproduced with permission from *J. Electrochem. Soc*. **2004**, *151*, C614–C626. © 2004, The Electrochemical Society].

Despite the first observation of porosity evolution in np-Au during early 1990s (an early report of pattern formation during dealloying dates back to 1920s [23]), the actual mechanisms that played a pivotal role were not well-studied until more than a decade later. Erlebacher et al. used an elegant kinetic model to describe the nanoporosity formation in a Au-Ag alloy, using only diffusions of silver and gold, and a dissolution of silver [24,25]. They suggested that spinodal decomposition arranges the gold atoms in two-dimensional clusters at the surface of the alloy, as silver is dissolved. Through this process, the new underlying alloy is constantly exposed to the electrolyte setting the length scale of the pore morphology. Figure 2 schematically summarizes the proposed mechanism.

Several other studies focused on porosity evolution, such as geometric relaxation during dealloying and different binary alloy systems. Crowson *et al.* conducted atomic-scale simulations to show that overall geometric relaxation of nanoporous metals is primarily dominated by surface relaxation, followed by capillary effects [26]. A kinetic model was shown to capture the effect of different less-noble alloy constituents in porosity formation [27].

Although now a general understanding of porosity evolution exists, further research is necessary to elucidate the effects of stress generation during dealloying in structures under different mechanical constraints and the effects of multiple alloy constituents in pore morphology.

### 3.2. Alloy Preparation

Conventional synthesis of np-Au requires an initial alloy consisting of at least a noble constituent and a chemically-dissolvable constituent. The Au-Ag system has been the most commonly used alloy for synthesizing np-Au, due to (i) availability of etching techniques with high selectivity for silver; (ii) complete solid solubility across all compositions; and (iii) mechanical compatibility (e.g., similar yield stress, thermal expansion, *etc.*). Other binary and ternary alloy systems have also been explored, including Au-Zn [28], Au-Ni [29], Au-Al [30], and Au-Au-Pt [31]. More experimental and theoretical studies are necessary to investigate the relationship between alloys constituents and resulting morphologies.

Numerous techniques have been developed to produce the most abundantly used precursor, the Au-Ag system. Many groups use bulk Au-Ag ingots, rods, and sheets produced by hammering, folding, or melting alloy constituents and subsequent dicing, rolling, or drawing into various sample sizes and shapes [32,33,34,35,36]. Such millimeter-scale structures are generally obtained by melting and mixing two pure alloy precursors, annealing to relieve residual stress, and machining to produce desired dimensions. An inexpensive source of thin Au-Ag alloy has been Monarch “white-gold” leaves sold at art stores for decorative purposes [37]. The leaves are usually 10 cm by 10 cm and the thickness typically ranges between 100 nm to 200 nm. Several groups have used them for mechanical testing [38,39], fuel cells [22], and fabrication of highly flexible conductive bilayer membranes [40]. The elemental composition of the alloy leaf is annotated by its karat value (e.g., 12-kt white gold has equal weights of gold and silver).

The aforementioned methods are not directly compatible with conventional microfabrication processes, thus preventing their straight-forward integration and limiting their versatility. Ideally, the alloy should be deposited by means of sputtering, evaporating, or electroplating to benefit from the established microfabrication technology. Researchers have developed various methods to take advantage of these deposition techniques. Ji *et al.* fabricated np-Au wires by simultaneously electroplating a single-phase Au-Ag alloy on porous alumina substrates using potassium cyanide solutions of gold and silver, and subsequently releasing them by dissolving the alumina template [41]. It is possible to produce relatively thick films (tens of microns) and still produce micro-patterns with electroplating. However, electroplating frequently suffers from repeatability issues and it is difficult to produce crack-free thick films or intact high-aspect ratio np-Au structures. Simultaneous magnetron-sputtering of alloy constituents offers precise control over deposited film properties, including composition, residual stress and grain size, by adjusting process gas composition, pressure, flow, substrate temperature, gun powers, and ion acceleration towards the substrate. Numerous groups have used this technique extensively to deposit blanket thin films and micro-patterns [19,42,43,44,45]. A less commonly used method is the co-evaporation of gold and silver pebbles with an electron beam [4], which offers good control of film thickness and alloy composition, yet lacks the ability to control process gas ambient, as depositions are invariably carried out under high vacuum. Unfortunately, it is unpractical to deposit thick films by sputtering or evaporation, due to low deposition rates, wasted material, and ensuing expenses. It is important to note that Au-Ag films alone are not sufficiently adherent to silicon or glass substrates. Traditionally, depositing a thin layer of chrome and gold prior to the deposition of the actual alloy promotes adhesion of the final np-Au film. Most delamination problems during dealloying are due to the absence of the adhesive layers and in part due to residual stress accumulation due to volume contraction.

### 3.3. Dissolution Methods

Nitric acid is commonly used to dealloy Au-Ag in order to produce np-Au and has the advantage of circumventing the use of electrochemical dissolution circuitry. However, this comes with the trade-off of diminished control of pore size. Alternatively, application of an anodic potential during dealloying in perchloric acid increases the silver dissolution rate compared to the gold diffusion rate, and hence results in finer porosity. The latter method has a greater number of controllable parameters, such as electrolyte temperature, critical potential (potential where the less noble constitute begins to dissolve), electrolyte composition, *etc.* Several groups have investigated key parameters during this process [33,46,47,48,49]. Dursun *et al.* showed that a halide addition into the dealloying electrolyte leads to a reduction of the critical overpotential as a result of competition between the rates of Au surface diffusivity and Ag dissolution modulated by halides [47]. Synder *et al.* demonstrated a dissolution strategy using silver nitrate solutions at neutral pH and speculated that silver is leached by the “acidic” accumulation of protons during surface oxide formation and water dissociation [49]. This method, along with the dissolution of Al from Au-Al alloys in saline solutions under potential-control [30], promises safe techniques for np-Au production. 

Even though dissolution with a prescribed potential offers higher control over pore morphology, there are instances, where dealloying with a potential is not practical. In such cases, dealloying in nitric acid is preferred [19,38,40,42,43,50,51]. For example, micro-patterns that are not electrically connected cannot be dealloyed in this manner and are usually immersed in nitric acid. Similarly, white gold leaves are generally dealloyed without a potential. In order to produce a np-Au leaf, the white gold leaf is transferred over a bath of concentrated nitric acid using a carbon roller or brush. The surface tension of the liquid evenly spreads the leaf over the surface and dealloying proceeds. When the leaf no longer changes color (~30–60 minutes), it can be collected with the roller [37] or a substrate such as a silicon wafer [40] for further use. Unfortunately, np-Au leaves are highly fragile and very difficult to handle.

### 3.4. Micropatterning Techniques

Most studies on np-Au employ machined millimeter-sized ingots, discs, or foils, which have been instrumental in fundamental understanding of morphological, electrochemical, and preliminary mechanical properties of np-Au. However, macro-scale fabrication approaches have some shortcomings, including: (i) difficulty in interfacing np-Au structures with electronics or microfluidics for miniaturized sensor platforms; (ii) limited ability conduct experiments that can survey different sample properties in parallel; and (iii) reduced potential in revealing phenomena unique to np-Au sample geometries approaching pore dimensions. The advent of microfabrication techniques in np-Au research has been expanding the experimental and application potentials significantly by mitigating some of these challenges. 

**Figure 3:**
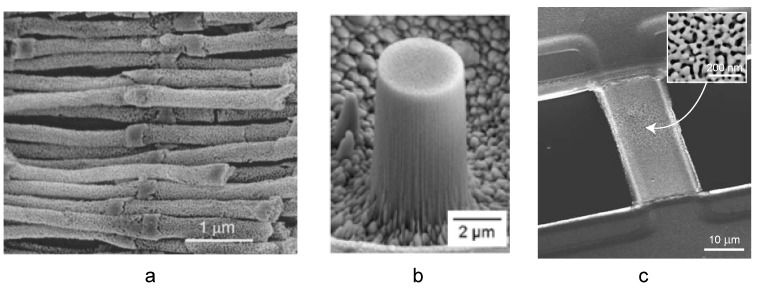
Examples of microfabricated np-Au structures: (a) nanowires produced by electroplating on a porous alumina template [Reproduced with permission from *Appl. Phys. Lett*. **2002**, *81*, 4437–4439. © 2002, American Institute of Physics]; (b) nano-pillar fabricated by focused ion beam [Reproduced with permission from *Appl. Phys. Lett*. **2006**, *89*, 061920. © 2006, American Institute of Physics]; and (c) freestanding beam fabricated by photolithography. [Reproduced with permission from *Acta Mater*. **2007**, *55*, 4593–4602. © 2007, Elsevier].

Figure 3 illustrates some examples of microfabricated np-Au structures. The nanowires were synthesized by electroplating the Au-Ag alloy onto a porous alumina template, dissolving the alumina template in KOH solution, and *in situ* dealloying of released Au-Ag nanowires (Figure 3a) [50]. The resulting np-Au nanowire diameter was in the range of ~200 nm and defined by a combination of the porous alumina pore diameter and the volume shrinkage during dealloying. Typical pore and ligament sizes of np-Au nanowires produced from Au_0.18_Ag_0.82_ (all alloy compositions in the manuscript are atomic fractions) were both 20–30 nm; while Au_0.32_Ag_0.68_ nanowires led to larger pore and ligament sizes, 10–40 nm and 35–50 nm respectively, due to larger gold content. The np-Au nanowires revealed significant impedance changes upon adsorption of thiolated-alkyl chains owed to comparable dimensions of nanowire diameter and pore sizes [5], exemplifying the potential of miniaturization in probing unique nanoscale phenomena. Liu *et al.* expanded the np-Au nanowire concept to fabricate a flow-through porous membrane [52]. They electroplated Au-Ag into the channels porous alumina templates (~45–210 nm-diameter channels, ~10^8^–10^10^ channels/cm^2^) and later dealloyed the structures to form np-Au nanowires (pores and ligaments with ~20 nm characteristic size) within the channels. This is a promising method to fabricate flow-through catalysts or molecular sieves; however, the pressure-flow relationships with respect to membrane architecture need to be established in order to implement this structure in such applications.

An enabling technology for probing the mechanical properties of materials has been the fabrication of nano-pillars (Figure 3b). These structures are frequently fabricated using a focused ion beam (FIB) instrument to carve out pillars from bulk materials such as dealloyed Au-Ag ingots [34,35]. Typical pillar dimensions are 1–8 µm and aspect ratios are 2–3. As seen in the figure, accelerated ions lead to striations on the side surface of the pillars. It is speculated that such modifications on the surfaces of micron-size structures may have an effect on mechanical properties via arresting dislocations or creating dislocations. There has been limited research studying the potential effects of FIB on metallurgical and mechanical properties of pillars [53].

**Figure 4:**
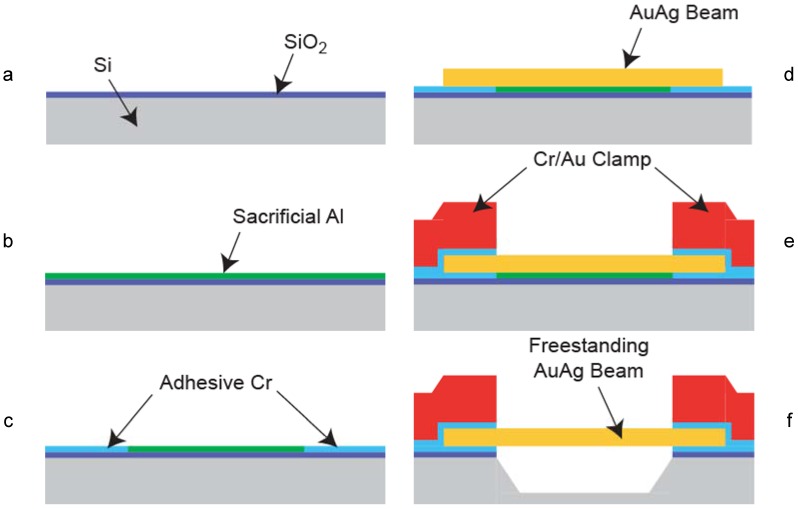
Schematic of freestanding Au-Ag beam fabrication: (a) ~80 nm-thick thermal SiO_2_ is deposited onto a p-type 50 mm Si (100) wafer; (b) ~60 nm-thick Al is sputter-deposited to serve as a sacrificial layer; (c) planar foundation layer is produced by etching and photolithographic-patterning Cr pedestals for adhesion; (d) ~800 nm-thick Au_0.3_Ag_0.7_ (atomic fraction) beam is sputtered-deposited through a photoresist micro-stencil layer; (e) beam is clamped onto the substrate by sputter-depositing Cr (~50 nm-thick) and Au (~500 nm-thick), and subsequent electroplating of ~3 µm-thick Au; and (f) beam is released by consecutively etching Al, SiO_2_, and Si, and critical-point drying.

Powerful sensor applications of np-Au will likely find use in handheld devices that can identify minute amounts of pollutants in water sources in remote settings or quantify disease-markers in blood for point-of-care applications. Such technologies will require successful integration of np-Au sensors with peripheral electronics or microfluidic channels, where the ability to produce np-Au sensor components with conventional microfabrication techniques is crucial. Photolithographic patterning techniques, employing photo-sensitive polymer coatings as stencil masks or etchant-resistant masks (illustrated in Figure 4), are essential in producing np-Au patterns that can interface with peripheral interconnects of microprocessors. 

Microfabrication of freestanding MEMS-like np-Au structures, illustrated in Figure 3c, also assisted the study of mechanical and morphological properties under different mechanical constraints [42,43,54]. In order to achieve this, a combination of bulk- and surface- micromachining techniques were used to produce an array of freestanding Au-Ag alloy beams. Figure 4 outlines the method for producing Au-Ag beams (which are used for creating np-Au beams), displayed in Figure 3c. The ability to precisely pattern thousands of ~1 µm-thick beams with 5 to 50 µm widths and 50 to 500 µm lengths should enable studies elucidating size effects on porosity evolution and resultant mechanical properties. In addition, precise thickness control offered by thin film deposition technology can create beams that are only a few pores thick, thereby allowing the study of quasi-two-dimensional ligament networks. As an alternative to sputter-deposition, Lee *et al.* followed a different strategy to prepare freestanding microbeams [38,55]. They laminated a Au-Ag leaf onto a silicon wafer, patterned it by dry etching through nickel hard mask, and subsequently dry etched the silicon wafer to suspend the dog-bone shape structures.

**Figure 5:**
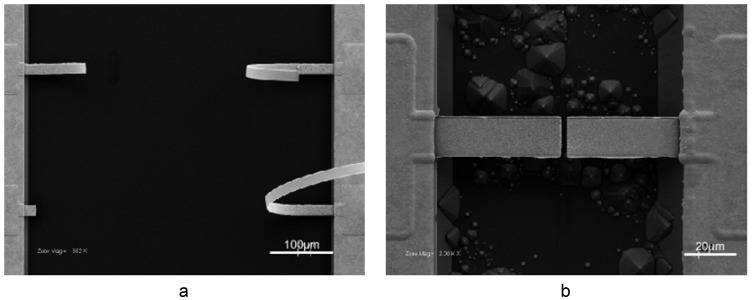
(a) When Au-Ag cantilevers are dealloyed, they warp significantly. (b) np-Au cantilevers can be produced by cutting np-Au beams with focused ion beam.

While it is possible to fabricate np-Au beams from micro-patterned Au-Ag beams (with the method to be described in Section 3.6), micro-patterned Au-Ag cantilevers warp catastrophically during dealloying, probably due to asymmetric dissolution rates, and hence stress generation, on opposite surfaces of the beams (Figure 5a). However, it is possible to produce np-Au cantilevers by cutting np-Au beams with FIB (Figure 5b) [42].

Microfabrication techniques are vital tools for high throughput sample production where a matrix of different structures (e.g., beams, pillars, stripes) with different properties (e.g., geometry, thickness, alloy composition) can be patterned on a single substrate. This allows for a highly parallel survey of alloy and final np-Au properties (e.g., mechanical, morphological, optical, electrochemical). We envision this technology to greatly facilitate optimization of np-Au properties for specific applications, most notably for sensors.

### 3.5. Morphology Modification

**Figure 6:**
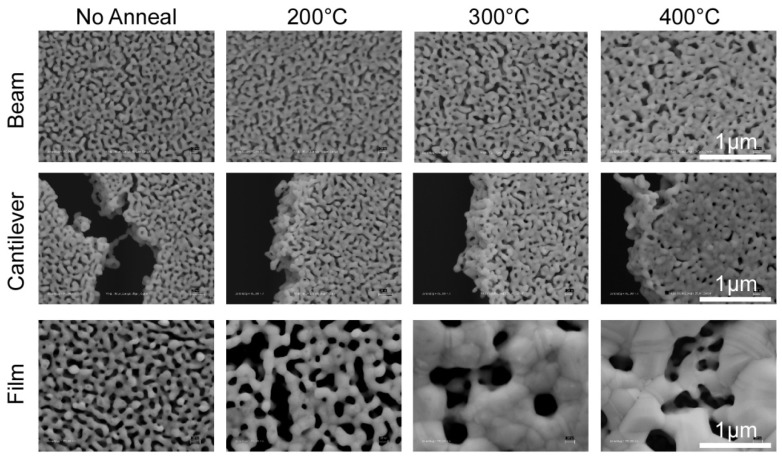
Scanning electron micrographs of np-Au structures under different mechanical constraints. [Reproduced with permission from *Acta Mater*. **2007**, *55*, 4593–4602. © 2007, Elsevier].

The most frequently used method to modify morphology has been thermal treatment, which leads to coarsening of pores and ligaments [17,19,42,45,56,57,58,59]. The underlying mechanism of thermal coarsening is in part due to the increase in surface diffusion of gold atoms leading to ligament growth and thereby increasing the average pore size. Using thermal treatment, pore and ligament sizes can be controlled within a wide range of 5 nm to a few microns. It is also striking that thermal coarsening preserves the starting pore structure, it simply exaggerates the characteristic features (compare film morphology for “No Anneal” and “200 ºC” in Figure 6). Another approach has been to vary the initial alloy composition in order to produce different morphologies. Scanning electron microscope images of np-Au surfaces obtained from different alloy concentrations revealed that higher initial silver content resulted in higher porosity. The authors note that 35% gold (atomic weight) is the parting limit for the Au-Ag system, that is, no porosity evolution is observed for higher gold content [60].

One method to prevent undesired coarsening of np-Au films is to prevent surface diffusion of gold. This has been effectively demonstrated with the addition of platinum to the initial alloy [31] or deposition of small amount of platinum onto the np-Au film [20]. Stabilization of the pore structure greatly enhances the usability of this material in high temperature conditions that lead to pore coarsening such as fuel cells [20]. Adsorption of a residual layer of oxygen during dealloying serves a similar stabilizing purpose [15]. The use of ternary alloy constituents is a promising method for not only stabilizing np-Au films, but potentially creating more complex pore structures.

Dursun *et al.* suggested a relationship between halides in dealloying electrolyte and the final nanoporous morphology [47]. They have observed that the size scale of pores increased with the addition of halides, with almost an order of magnitude increase (~ 8 nm to 67 nm for np-Au produced from Au_0.35_Ag_0.65_) with KI-containing electrolytes.

The mechanical constraints, and hence stress evolution in np-Au structures exposed to thermal treatment, result in profound differences in porosity evolution [42], as shown in Figure 6. The porosity of all three structures decreased (from ~30% to ~15%) as the thermal treatment temperature increased. Conversely, average pore size decreases insignificantly for beams and cantilevers, while the blanket films exhibited non-monotonic increases with increasing temperature. Beams developed micro-cracks in response to accumulation of tensile stress, while cantilevers contracted as their pores vanished. Films displayed the evolution of large clusters of gold and significant pore coalescence (accompanied by reduction in film thickness) with increasing temperature. Differences in morphology are also evident between beams, cantilever, and blanket films that were not exposed to thermal treatment, suggesting that it is probable that mechanical constraints (and resultant stress states) lead to morphological changes in porosity evolution during dealloying. The effect of mechanical constraints in morphology evolution requires further research, and can benefit greatly from molecular simulations. An interesting experiment may involve real-time observation of porosity evolution under thermo-mechanical stress using a scanning electron microscope with a heated stage to probe routes of coarsening.

Ding *et al.* developed an interesting approach to create np-Au with multimodal pore size distribution, where they electroplated a secondary Au-Ag alloy (with an alternate composition) onto an existing np-Au template and subsequently dealloyed it to reveal a composite pore morphology [61]. Hakamada *et al.* compared the effects of thermal treatment and acid treatment in coarsening of pore and ligament sizes of np-Au sheets [56]. They observed that thermal treatment resulted in uniform coarsening, while acid treatment in concentrated HCl surprisingly produced strands of porous regions surrounded with solid walls of gold. They suggested that different gold migration routes during treatments may be responsible for different coarsening schemes. Others have shown that prolonged dealloying in nitric acid led to ligament coarsening similar to coarsening observed during thermal treatment, probably due to similar underlying mechanisms involving increased gold surface diffusion [37]. It is interesting that different acids (*i.e.,* HNO_3_ and HCl) result in remarkably different morphologies and the underlying mechanisms are unclear.

### 3.6. Fracture and Remedy

A major problem in synthesis of np-Au films is the formation of voids and/or cracks in films, hindering their application as functional coatings. A significant proportion of the crack formation in np-Au films is due to volume contraction during dealloying. This phenomenon has been observed by different groups [42,50,62]. Parida *et al.* suggested that the volume reduction may be a result of small scale plastic deformation due to homogeneous slip in small ligaments or by climb of lattice dislocations [62]. The volume contraction phenomenon should be more carefully studied with the aid of molecular simulations, taking key conditions into account, such as electrolyte type, temperature, sample dimensions, and dealloying potential.

**Figure 7:**
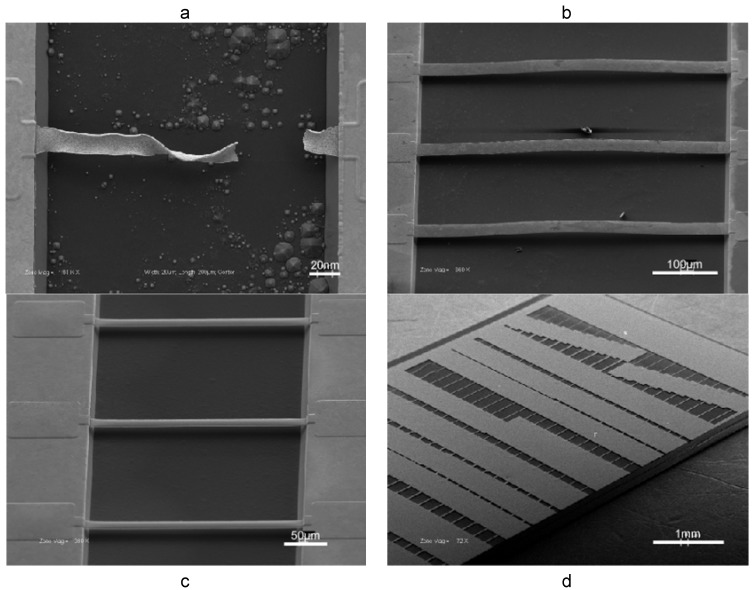
Illustration of fracture and remedy in np-Au: (a) freestanding doubly-clamped beams catastrophically fail during dealloying due tensile stress accumulation as a result of volume contraction; (b) rapid thermal treatment permanently buckles Au-Ag beams; (c) buckling strain offsets volume contraction and intact np-Au beams are realized; (d) a typical chip containing ~200 intact np-Au beams of various dimensions. [Reproduced with permission from *Acta Mater*. **2008**, *56*, 324–332. © 2008, Elsevier].

Shrinkage during dealloying becomes especially problematic in fabrication of constrained microstructures, such as doubly-clamped beams [42]. Figure 7a is an SEM micrograph illustrating catastrophic failure of a microbeam during dealloying. A novel solution to this problem is the rapid thermal treatment of solid Au-Ag alloy microbeams, resulting in their permanent buckling [43], as shown in Figure 7b. The underlying mechanisms of how rapid thermal treatment leads to a permanently buckled beam shape are multifaceted. A complex interplay of work hardening, thermal expansion, and changes in alloy microstructure due to thermo-mechanical stress likely result in permanent beam buckling. Regardless, the buckled shape produces a positive strain to offset shrinkage during dealloying, thereby mitigating tensile stress accumulation during dealloying and producing intact np-Au beams (Figure 7c). The outlined process enabled the fabrication of hundreds of intact np-Au beams (~3600 beams per 50 mm wafer) with close to 100% fabrication yield (Figure 7d). The beam dimensions ranged between 5 to 50 μm in width and 10 to 500 μm in length. Systematic variation of the thermal treatment temperature of the alloy exposed a correlation between the maximum amplitude of permanent beam buckling (buckling amplitude increases monotonically with temperature up to 350 ºC) and percentage of microbeams that survived dealloying without fracture.

**Figure 8:**
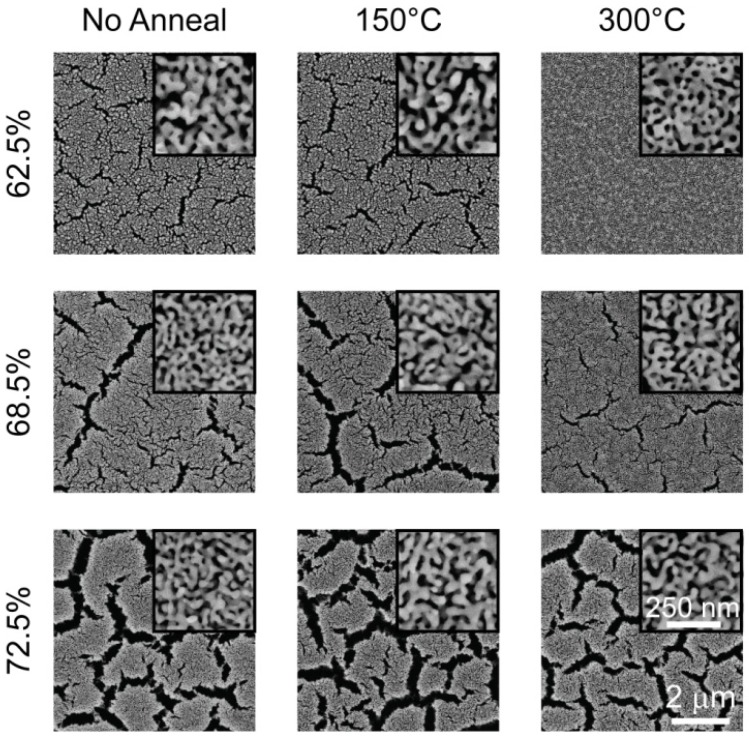
Micrographs of the np-Au films (with various initial Ag content) created by dealloying the thermally treated Au-Ag films. The larger images and the insets exemplify the typical crack formations and pore morphology, respectively. [Reproduced with permission from *Scripta Mater*. **2009**, *60*, 435–438. © 2009, Elsevier].

Similar crack/void formations are evident in blanket np-Au films on rigid substrates [19,44], as well as micropillars [35]. Methods to alleviate these formations include low-temperature dealloying [63] and multi-step dealloying techniques [64]. Thermal treatment of the blanket alloy film non-monotonically reduced void formation [44]. Figure 8 illustrates the effect of thermally treating a Au-Ag blanket film prior to dealloying. While there were not significant changes in nanopororous structure, thermal treatment generally reduced cracks. It is plausible that thermal treatment leads to work hardening of the alloy and increases the ultimate strength of ligaments; that is, if the failure of a critical number of ligaments leads to crack/void formation, stronger ligaments will in turn reduce crack formation in blanket films.

Other groups have emphasized the importance of microstructural length scale of np-Au and ligament size in ductile-brittle transitory behavior of np-Au, as well as its fracture strength [34,58]. Barnes *et al.* observed a phenomenon where complete intergranular or transgranular fractures of Au-Ag wires and foils can be induced by applying a small tensile stress to structures during dealloying (or soon after dealloying when still wet) [32]. It is possible that a similar mechanism may be responsible for rapid crack propagation in thin films and freestanding structures during dealloying. In addition, it is likely that dealloying parameters, such as electrolyte composition, dealloying potential, and temperature, play an important role in the scale of volume contraction. Systematic studies on this can provide insight into developing effective strategies to control shrinkage during dealloying.

## 4. Characterization

Researchers have utilized numerous existing methods and novel techniques for the analysis of alloys and np-Au structures. In this section, we provide a review of current techniques of characterization and summarize key findings.

### 4.1. Mechanical Characterization

**Figure 9:**
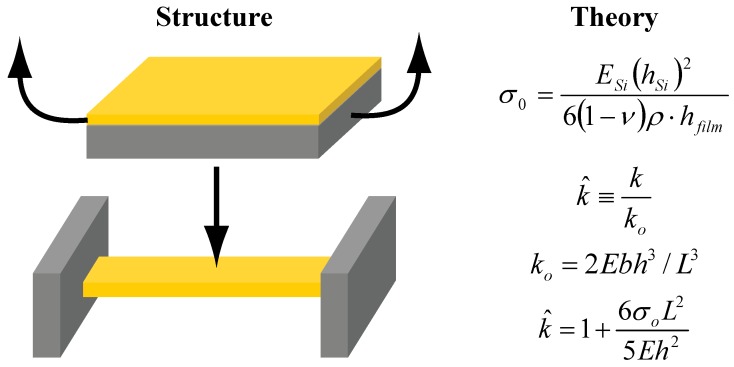
Illustration of common mechanical characterization methods to measure residual stress. In the beam residual stress calculation, $\overset{˰}{k}$ is the ratio of measured beam stiffness *k* to theoretical beam stiffness *k_0_*, *E* is beam elastic modulus, *b* is beam width, *h* is beam thickness, *L* is beam length, and *σ_0_* is the beam residual stress. For blanket film residual stress calculation, *E_Si_* is elastic modulus of silicon wafer, *h_Si_* is silicon wafer thickness, *υ* is Poisson’s ratio of silicon wafer, *ρ* is measured radius of curvature of silicon wafer, and *h_film_* is the film thickness.

Residual stress in structures is of great importance, in blanket films and miniature structures alike. Compressive residual stress may lead to delamination of blanket films and buckling of freestanding structures. Conversely, tensile stress is the main culprit for cracking and for some delamination. Figure 9 displays common techniques to measure residual stress in films and beams. Wafer curvature measurement methods are practical in obtaining residual stress in films deposited over stiff substrates, where Stoney’s equation is used to relate the radius of curvature to residual stress in the blanket film (Figure 9) [65]. Residual stress measurements in freestanding beams, however, are more complicated, and stress is extracted from the ratio of measured beam stiffness to theoretical beam stiffness (Figure 9) [66]. This treatment requires the elastic modulus value of the beam, which can be extracted from stiffness of stress-free cantilevers. For both residual stress and elastic modulus calculations, a nanoindentation instrument (along with a novel measurement protocol [67]) is used to conduct beam-deflection measurements to obtain beam stiffness. Another method to determine residual stress is to measure changes in lattice constants (strain due to residual stress) by X-ray diffraction and convert these changes to residual stress values [68].

Thermal annealing of Au-Ag blanket films on silicon wafers led to temperature-dependent increases in residual stress (from ~100 MPa for as-dealloyed state to ~350 MPa for thermal treatment at 300 ºC). This is attributed to work hardening of the alloy during thermal cycling-related compression-tension [44]. Regardless of the residual stress in the alloy, the stress was mitigated through void formation in the final nanoporous film, reducing the residual stress in np-Au film to less 20 MPa [44]. In addition, the initial alloy composition did not have a significant effect on the residual stress in the final gold structure, or at least produced stress values below the sensitivity of the wafer curvature apparatus. However, thermal treatment of np-Au blanket films led to a dramatic increase in residual stress (from ~20 MPa for as-dealloyed state to ~80 MPa for thermal treatment at 400 ºC) [42]. Residual stress in freestanding beams (produced from Au_0.4_Ag_0.6_ beams) also increased with thermal treatment temperature (from ~10 MPa for as-dealloyed state to ~30 MPa for thermal treatment at 400 ºC); however, the residual stress was generally lower compared to blanket films, in part due to minimized substrate interaction with the beam, hence more freedom to relax. Lee at al. measured residual stress of microfabricated np-Au beams (7 μm-long, 300 nm-wide, and 100 nm-thick) synthesized from white gold leaves (Au_0.37_Ag_0.63_) and obtained ~65 MPa [38]. It is difficult to compare the residual stress values from the beams produced with significantly different microfabrication techniques and dimensions [38,43]; however, it is plausible that smaller beam cross-sections and no prior thermal treatment (no permanent deformation of alloy beam prior to dealloying, hence no stress mitigation) produced by Lee *et al.* increases the yield stress of np-Au beams, thereby leading to higher residual stress values compared to the beams fabricated by “permanent buckling” approach. This is a striking example of how different experimental conditions can lead to large variations in residual stress in np-Au freestanding structures. Nevertheless, there is limited literature on residual stress of np-Au structures.

**Figure 10:**
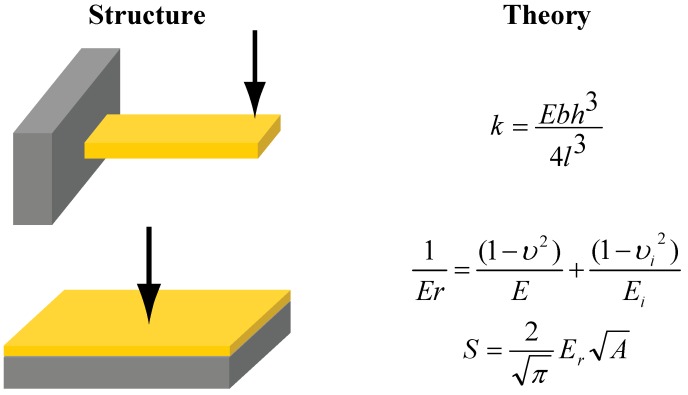
Illustration of various mechanical characterization methods and structure employed. In the cantilever elastic modulus calculation, *E* is elastic modulus, *b* is beam width, *h* is film thickness, *l* is distance between point of measurement and beam pivot point, and *k* is measured beam stiffness. In the blanket film elastic modulus calculation, *υ* and *υ_i_* are Poisson’s ratios of film and indenter tip respectively, and *E* and *E_i_* are elastic moduli of film and indenter tip respectively. In the calibration equation, *S* is measured stiffness, *A* is tip contact area, and *E_r_* is reduced elastic modulus. Calibrations are usually done on silica standards.

Elastic modulus is another important mechanical property and is required to calculate residual stress in freestanding beams, as seen in Figure 9. Material selection is of utmost importance, especially, from chemo-mechanical sensors’ point of view, wherein decreased film stiffness was shown to increase sensor performance [69]. Figure 10 summarizes common techniques to measure elastic modulus of beams and films. Nanoindentation is perhaps the mostly commonly used technique to determine elastic modulus of thin films and bulk samples. Biener *et al.* used two different nanoindenter probes (*i.e.,* conospherical and Berkovich) to determine elastic modulus (~11 GPa) of 0.5 mm-thick np-Au samples with ~46% porosity and ~100 nm ligaments [34]. Elastic modulus values of ~20 GPa were measured on micron-thick np-Au films (~35% porosity and ~100 nm ligament size) prepared from sputtered Au-Ag thin films using the equation displayed in Figure 10 [42]. This method was adopted from Oliver and Pharr, where small continuous load-unload curves were captured by imposing small oscillations on the tip during ramp loading [70]. With this technique, known as continuous stiffness measurement (CSM), it was possible to measure elastic modulus as a function of depth without loading and unloading to each depth.

As for measurements on freestanding np-Au beams, Lee *et al.* extracted measured elastic modulus of ~9 GPa on dog-shape beams described earlier [38], while values in the range of 10–20 GPa were obtained from beams fabricated using a different method [42,54]. These measurements employed beam-deflection measurements using either nanoindenters or atomic force microscopes. Elastic modulus of np-Au beams with different pore sizes (morphology modified by thermal treatment) was measured by beam-deflection measurements using a nanoindenter. Freestanding structures have the unique ability to minimize the substrate-film interaction, thereby allowing more accurate extraction of film properties. The mechanical properties of freestanding microbeams and blanket films were contrasted to see the effect of mechanical constraints. Freestanding np-Au cantilevers, produced by cutting np-Au doubly-clamped beams with FIB, are stress-free, and their measured stiffness can be used to extract their elastic modulus using equation displayed in Figure 10. Through thermo-mechanical experiments involving Au-Ag and np-Au MEMS-like structures and blanket films, it is possible to control elastic modulus, residual stress, and pore morphology somewhat independently. Generally, thermal treatment of Au-Ag structures non-monotonically reduced crack formation and did not have a significant effect on pore morphology and consequently on elastic modulus. This technique commonly reduced tensile residual stress in doubly-clamped np-Au beams and did not have a noticeable effect on residual stress of np-Au blanket films. 

The emergence of compression tests on np-Au micropillars contributed more elastic modulus values to the current literature. Volkert *et al.* measured elastic modulus (~7–12 GPa) from the unloading segments of compression tests on nano-pillars with ~15 nm ligament sizes [35]. In addition to the micropillar studies, Mathur *et al.* collected stress-free np-Au foils produced from alloy foils on elastomer blocks and captured characteristic buckling wavelength when the block is uni-axially compressed [39]. They processed this data to calculate elastic modulus of 100 nm-thick np-Au foils with different ligament sizes (5 nm to 40 nm). The elastic modulus decreased exponentially from ~40 GPa to ~5 GPa with increasing ligament size.

**Figure 11:**
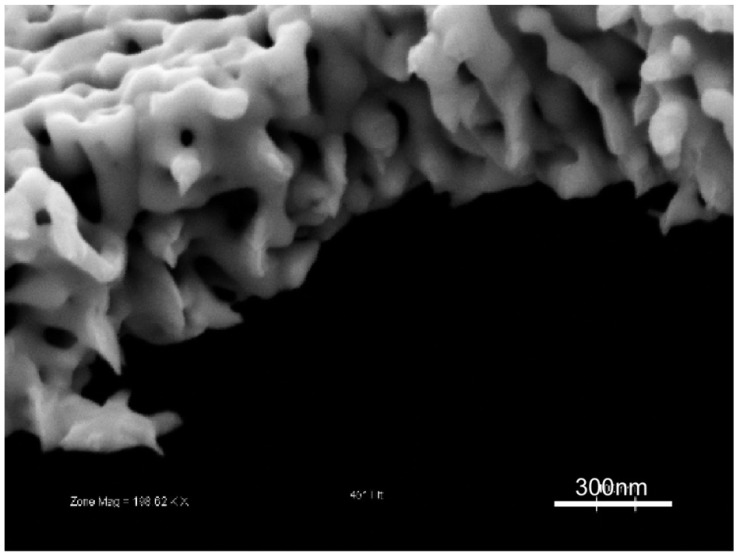
A high magnification SEM image of a fractured beam illustrating the ductile necking of ligaments. [Reproduced with permission from *Acta Mater*. 2007, *55*, 4593–4602. © 2007, Elsevier].

Most groups focused on yield stress and strength of np-Au, with the motivation of creating a low-density high-strength material. While np-Au films are notoriously brittle in macro scale, both SEM and TEM studies showed that ductile necking in severed ligaments demonstrate the ductile nature of np-Au [42,54,58,71]. Figure 11 illustrates such ductile necking evident on the edge of a fractured beam. Interestingly, Senior *et al.* demonstrated that np-Au strips can withstand much higher reversible mechanical deformations without fracturing when wet than when dry [33].

The majority of yield stress measurements utilized nanoindentation of bulk np-Au samples or compression tests on micropillars. Biener *et al.* performed nanoindentation studies on np-Au samples with ~42% porosity and ~100 nm ligaments and extracted yield stress of the porous network from hardness measurements to be ~145 MPa [34]. They noted that the Gibson-Ashby’s scaling laws for porous materials (*i.e.,* relationship between physical and mechanical properties [72]) under-predicted the yield stress of the porous network by an order of magnitude if macroscopic yield stress of gold (~200 MPa) is used. They argued that the nanometer-scale of ligaments and their yield stress approaches the theoretical yield strength of gold (~1.5 GPa), and cannot be adequately predicted using macroscopic mechanical properties of bulk polycrystalline gold. Similar studies produced supporting results [36]. A general observation with micropillars has confirmed the studies of bulk-nanoindentation, in which the yield strength increased with decreasing pillar diameter. This has also been observed by Greer *et al.* in compression studies of compact Au posts [73], possibly due to dislocation starvation, as suggested by the authors [74]. Volkert *et al.* performed compression tests on micron-sized np-Au nano-pillars with ligament diameters of ~15 nm and ~36% porosity. Using foam scaling laws, they predicted that the 15 nm ligaments yield around ~1.5 GPa, which is close to the theoretical strength of gold [35]—in agreement with the observations of Biener *et al.* [34]. They compared this value to gold samples up to ~10 µm length scales and noted that the yield stress is inversely proportional to approximately square root of characteristic sample size. Hodge *et al.* conducted a comprehensive study on scaling equations of porous foams with various ligament sizes (10–900 nm) and porosities (20–42%), and reiterated that the yield strength is not only determined by porosity but also by ligament diameters [59]. They modified the Gibson-Ashby scaling laws and incorporated a Hall-Petch type relation for the observed length-scale dependent strengthening. It is important to take into consideration that the mechanical properties (*i.e.,* elastic modulus, yield stress) of FIB-prepared micropillars with small volume-to-peripheral surface area may be dominated by the surface damage [53]. Others employed deflective-tensile testing of micron-sized freestanding beams to reach similar conclusions regarding the unexpectedly high yield stress of np-Au ligaments (~1.45 GPa) [38]. Jin *et al.* reviewed the current literature stating that yield stress increases with decreasing ligament size and contrasted this notion with their studies on crack-free np-Au samples (prepared at high electrolyte temperature and low dealloying potential) demonstrating much lower yield stress values predicted by others [75]. They suggested that the assumption that nanoindented film hardness is equal to yield stress is not true of np-Au and it overestimates yield stresses. 

The overarching significance elucidated by the mechanical characterization of np-Au foams has been to show that the mechanical properties (*i.e.,* residual stress, elastic modulus, yield strength) can be modulated via various techniques, which opens the door to development of advanced materials, such as low-density high-strength foams, as well as high-surface area low-elastic modulus coatings for chemo-mechanical sensors.

### 4.2. Geometric and Spectroscopic Measurements

**Figure 12:**
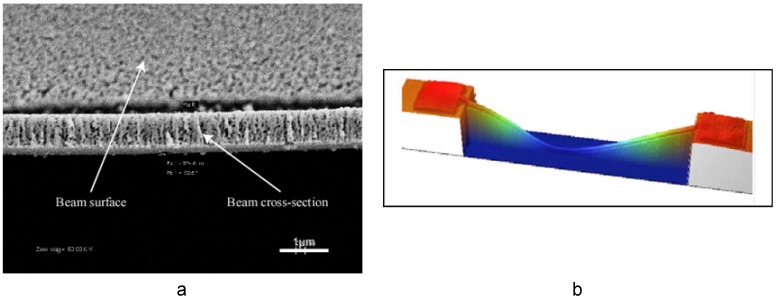
(a) FIB-prepared beam cross-section for thickness measurement. (b) A typical interferometric image of the buckled beam shape following rapid thermal treatment of freestanding Au-Ag beam.

Typically, blanket film thickness measurements involve the use of stylus-based profilometers or interferometric measurements. Stylus-based systems may deform the porous network unless low probe forces are employed. An alternative non-contact method of measuring film thickness is the use of white light interferometers, where two-dimensional interferometers (WLI) offer the additional flexibility in capturing surface topography such as roughness. Conventional measurement techniques are usually inadequate in determining thickness of freestanding microbeams. An *in situ* technique to prepare cross-sections of doubly-clamped beams using a combination of FIB and SEM resolves this problem [42]. Basically, a doubly-clamped beam is cut in the middle and a shallow incision is introduced away from the first cut to strain the segment between cuts upwards, thereby exposing the cross-section, as illustrated in Figure 12a . The beam thickness can then be easily measured using SEM images. Using this method, it was observed that thermal treatment of np-Au results in compaction, therefore an up to 30% decrease in film thickness at a treatment temperature of 400 ºC [42]. WLI systems are also useful in capturing beam shape (Figure 12b), in cases such as permanently deformed Au-Ag beams upon rapid thermal treatment [43]. Dynamic geometric changes, as in expansion of np-Au cubes when exposed to ozone [13], can be captured using a dilatometer in contact with the sample.

### 4.3. Material Analysis

Energy dispersive spectroscopy, where the material is excited by electron beams and the characteristic emission in high energy bands is measured, has been a vital tool in characterizing the elemental composition of initial alloy and residual constituents in the final nanoporous film [34,43]. Dealloying in nitric acid without applying a potential leads to final np-Au films with less than 3% (a.t) residual silvers [35,44]. Transmission electron microscopy (TEM) and X-ray diffraction studies have been valuable in studying grain and crystal microstructure of alloys and resultant np-Au samples [34,56,76]. Sun *et al.* reviewed *in situ* nanoindentation of np-Au in TEM, where ligament deformation at length scales at ~10 nm in ~75–300 nm-thick np-Au films and underlying mechanisms were investigated [77]. The authors observed load drops at indentation-rate-dependent intervals in nanoindentation of np-Au films. The authors suggested that the load drops may be due to collapse of the porous network or distinct plasticity events, which are initiated by dislocation nucleation.

### 4.4. Morphology Analysis

Total surface area, which made np-Au a very attractive material for sensor applications, can be estimated by gas adsorption methods [78] and electrochemical methods, such as electrochemical impedance spectroscopy [79,80]. Other electrochemical methods for determining surface area are oxidation/reduction of the gold surface and reduction of a monolayer of material (e.g., copper) on the gold surface, where the changes in charge are recorded for both methods [81]. In general, in order to determine the surface area by the electrochemical methods, measured electrochemical value (e.g., impedance, charge density) of a porous film is compared to that of a planar film and this ratio is used to calculate the surface area augmentation over a planar surface. Unfortunately, gas adsorption and electrochemical techniques are usually more effective in determining surface area than pore structure. Alternatively, electron microscopy, neutron scattering [82], or transmission electron tomography [83,84] can provide a more thorough inspection of the pore morphology. However, they also have their own shortcomings, such as limited applicability to samples that are not electron-transparent, lengthy sectional-image acquisition, and cumbersome statistical analysis. Many groups have extensively used scanning electron microscopy (SEM) for rapidly exploring porosity, pore size, and ligament size, due to their relative ease of use and applicability to a wide range of samples [44,58,85]. However, SEM studies rely on the assumption that two-dimensional surface morphology can be extrapolated to infer information about entire np-Au structure. While this assumption is not unreasonable in light of cross-sectional SEM images (obtained by cleaving samples or FIB) of np-Au structures displaying homogenous pore structure (Figure 1), the results lie somewhere between qualitative and strictly quantitative.

**Figure 13:**
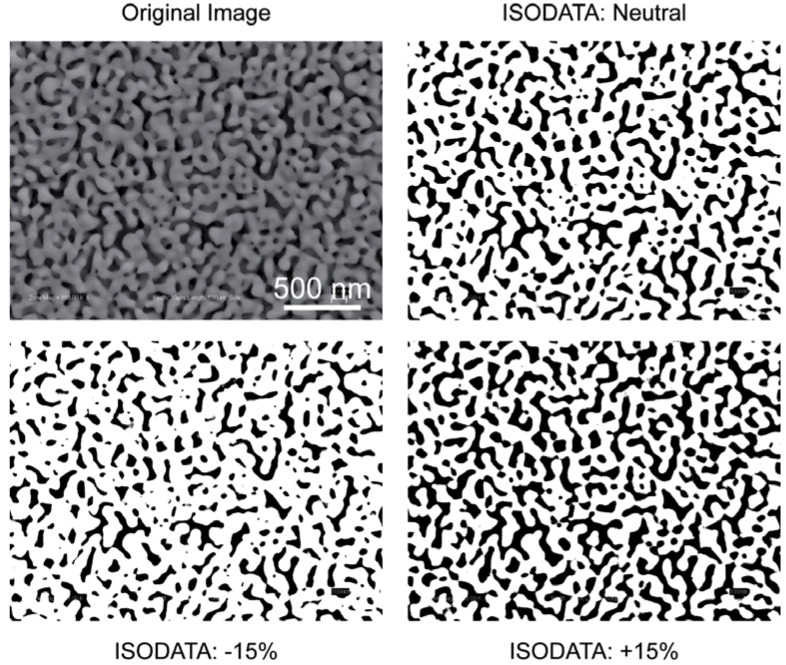
The percent coverage of pores (dark regions), as well as calculated values, depend on the thresholding level selected. For the example image above, the calculated percent porosity and average pore size values were 30% and 80 nm for the “neutral” case; 23% and 65 nm for the “−15%” case; and 39% and 110 nm for the “+15%” case.

Here, we will mostly focus on post-processing of SEM images for morphology analysis. Usually, the images acquired with SEM need to be post-processed to extract morphology parameters of interest. It is laborious to manually measure a statistically meaningful number of pores. It is more practical to use image analysis software for this purpose, such as ImageJ (NIH shareware, http://rsb.info.nih.gov/ij/index.html). Image processing of 2D morphologies has its own challenges as well. The first step of a typical image processing algorithm is the thresholding of a gray-scale image to produce a monochrome image. The central question here is picking a gray scale value (referred to as threshold value or segmentation value) that accurately represents the separation between dark regions (*i.e.,* pores) and light regions (*i.e.,* ligaments). In determining this, the imaging parameters such as working distance, brightness, contrast, and electron acceleration energy should be consistent for each image. The selection of the threshold value is highly prone to subjective bias, which can be circumvented (at least kept consistent from image to image) by using automatic thresholding algorithms, such as ISODATA (Iterative Self-Organizing Data Analysis Technique) [86]. The concept of thresholding has been a research interest on its own that has resulted in the development of various algorithms [87]. Figure 13 illustrates the effect of picking a thresholding value 15% above and below the automatically determined value. Higher threshold values (*i.e.,* bias towards defining lighter pixels as pores) results in over-estimation of porosity and average pore size. Another challenge is the irregularity of pore shapes, which complicates the determination of a characteristic pore size. The “watershed” algorithm partitions the images into regions that are roughly circles [88], which can then be used to calculated average pore diameters (also referred to as sizes). This additional step reduces the over-estimation of pore size. In conclusion, the digital image processing is a versatile method for morphology analysis, especially for comparative studies of samples (e.g., difference in np-Au morphology due to thermal treatment, dealloying conditions, initial alloy composition, *etc.*). For quantitative analyses, approaches such as transmission electron tomography may be appropriate.

## 5. Conclusions and Future Studies

We have presented a review of recent research in np-Au with an emphasis on fabrication and characterization techniques. Despite the rapidly growing knowledge in mechanical properties of np-Au, there are still unknowns with regard to fracture mechanics, the effects of alternate gold alloys on resultant pore morphology and mechanical properties, effects of sample geometry on pore evolution, and mass transport within the porous network. Here, we summarize some areas, in our opinion, that require further studies:

- Although now a general understanding of porosity evolution exists, it is necessary to study the effects of stress generation during dealloying in structures under different mechanical constraints and the effects of multiple alloy constituents in pore morphology.
- Identifying the relationship between alloys constituents and resulting morphologies, particularly for alloy systems with multiple constituents, can assist in expanding the repertoire of nanoporous gold with distinct pore structures and enable a multitude of applications.
- The coarsening effect of thermal treatment on porosity evolution is well known, but the dynamics of the process are probed mostly theoretically. An interesting experiment may involve real-time observation of porosity evolution under thermo-mechanical stress using a scanning electron microscope with a heated stage to provide insight into routes of coarsening.
- Film delamination and cracking limit the successful integration of np-Au as functional coating. Most delamination problems during dealloying are a result of the absence of the adhesive layers between the np-Au layer and the substrate, compounded by residual stress accumulation due to volume contraction. Characterization of the interface strength and optimization of fabrication variables may mitigate such issues, thereby enabling synthesis of intact np-Au structures.
- Volume contraction during dealloying is widely observed and there are several methods to blunt its scale; however, it should be more systematically studied with particular attention to the effects of key parameters, such as electrolyte type, temperature, sample dimensions, and dealloying potential.
- Flow-through np-Au catalysts and molecular sieves are promising applications; however, the pressure-flow relationships as well membrane strength with respect to membrane architecture need to be established in order to implement this structure in such applications.

The aforementioned questions will likely require the development of novel fabrication and characterization methods, as well as modeling approaches. We envision that combined efforts focusing on these issues will increase our understanding of np-Au and facilitate its implementation in a variety of applications. One such important application is the incorporation of np-Au for medical purposes, including immuno-sensors and neural electrodes. Such devices can be very powerful as point-of-care medical devices provided that their dependence our bulky external circuitry is prevented. In that aspect, we expect that microfluidic platforms would greatly benefit miniaturization of np-Au based sensors. The research at the interface of biology and np-Au is nascent. We foresee great potential in np-Au as a functional medical coating, which can be realized by research focusing on the biocompatibility of np-Au via tissue-material interaction studies. In addition, np-Au is a strong candidate for controlled drug delivery, due to its highly controllable pore morphology [89], which should be elucidated in terms of molecular elution characteristics from np-Au films. Non-medical applications of np-Au already exist. Future research will help delineate the range of contributions np-Au will have. Without a doubt, np-Au will remain an attractive material for fundamental and applied research alike.
